# Clinically relevant increases in the international normalized ratio and model of end-stage liver disease score by therapeutic doses of direct oral anticoagulants in patients with cirrhosis

**DOI:** 10.1016/j.rpth.2023.100052

**Published:** 2023-01-14

**Authors:** Ton Lisman, William Bernal, Jelle Adelmeijer, Pieter-Willem Kamphuisen, Sarah Bos, Robert J. Porte

**Affiliations:** 1 Surgical Research Laboratory, Department of Surgery, University of Groningen, University Medical Center Groningen, Groningen, the Netherlands; 2 Section of Hepatobiliary Surgery and Liver Transplantation, Department of Surgery, University of Groningen, University Medical Center Groningen, Groningen, the Netherlands; 3 Liver Intensive Therapy Unit, Institute of Liver Studies, Kings College Hospital, Denmark Hill, London SE5 9RS, UK; 4 Department of Internal Medicine, Tergooi Hospital, Hilversum, the Netherlands and Department of Vascular Medicine, Amsterdam Cardiovascular Science, Amsterdam University Medical Centres, University of Amsterdam, Amsterdam, the Netherlands; 5 Department of Gastroenterology, Treant Hospital, Emmen, the Netherlands

**Keywords:** anticoagulants, cirrhosis, end-stage liver disease, international normalized ratio, liver transplantation

## Abstract

**Background:**

Patients with cirrhosis are increasingly using direct oral anticoagulants (DOACs) in therapeutic doses for the treatment of portal vein thrombosis or for concomitant atrial fibrillation. DOACs may affect routine diagnostic tests of coagulation including the international normalized ratio (INR). The INR is a part of the model of end-stage liver disease (MELD) score, a validated score that predicts the mortality risk in patients with cirrhosis and is used to prioritize patients for liver transplantation. DOAC–induced increases in the INR may thus lead to artificial inflation of the MELD score.

**Objective:**

We studied the effect of DOACs on INR prolongation in patients with cirrhosis.

**Methods:**

We spiked plasma from 20 healthy individuals and 20 patients at the start of liver transplantation with DOACs in concentrations representing peak therapeutic levels. In addition, we studied INR increases in healthy controls and patients with mild cirrhosis who received the DOAC edoxaban for 1 week for study purposes.

**Results:**

In controls and patients, the INR increased by an *ex vivo* addition of a DOAC, and the INR increase in patients was proportional to the baseline INR values. The increase in INR translated to a median increase of between 3 and 10 MELD points, depending on the DOAC used. In controls and patients alike, the INR increased on the ingestion of edoxaban, which translated to an increase in 5 MELD points.

**Conclusions:**

Taken together, DOACs result in an INR increase that translates to clinically meaningful increases in MELD points in patients with cirrhosis, and precautions to avoid artificial inflation of the MELD score in these patients are warranted.

## Essentials

• Patients with chronic liver disease increasingly use direct oral anticoagulants (DOACs) to prevent or treat thrombosis.
• DOACs increase the international normalized ratio, a laboratory test used to prioritize patients for a donor liver.
• The model of end-stage liver disease score used for this prioritization process is substantially affected by DOACs.
• Measures to fairly and adequately prioritize waitlisted patients using DOACs are needed.

## 1 Introduction

Patients with cirrhosis may develop substantial alterations in their hemostatic system that paradoxically may be associated with both bleeding and thrombosis [1]. There is an increasing awareness of the thrombotic risk in patients with cirrhosis, notably the risk for venous thromboembolism (deep vein thrombosis and pulmonary embolism) [2] and the risk for portal vein thrombosis [3]. Patients may, therefore, be treated with anticoagulant drugs for the prevention or treatment of venous thromboembolism or portal vein thrombosis, and in addition, patients may receive anticoagulant drugs to prevent thrombotic complications related to atrial fibrillation.

Long-term anticoagulation in patients with cirrhosis may be accomplished using vitamin K antagonists, low-molecular weight heparin, or direct oral anticoagulants (DOACs). Because vitamin K antagonists are difficult to dose in patients with cirrhosis and an elevated baseline international normalized ratio (INR), and daily injections with low-molecular weight heparin are a substantial burden for the patient, DOACs are gaining popularity in the cirrhotic patients, although data on safety and efficacy are still scarce [4]. Despite this lack of firm evidence, recent clinical guidance documents state that DOACs are a reasonable treatment option in patients with Child A or B cirrhosis [5,6].

In patients with cirrhosis that become liver transplant candidates, the model for end-stage liver disease (MELD) score is used to prioritize patients on the waiting list. The MELD score is calculated using the following formula: 9.57 × log_e_ (creatinine) + 3.78 × log_e_ (total bilirubin) + 11.2 × log_e_ (INR) + 6.43. Because the MELD score includes the INR, the score is artificially inflated in patients receiving vitamin K antagonists. It has been previously proposed to use a simplified MELD score that does not include the INR to prioritize patients who use vitamin K antagonists [7]. Within Eurotransplant, the MELD score for waitlisted patients on vitamin K antagonists has to be calculated using the last value before starting vitamin K antagonists, or the vitamin K antagonists have to be stopped for at least 2 weeks to determine the current INR (https://www.eurotransplant.org/wp-content/uploads/2022/03/H5-ELAS-MELD-March-2022.pdf).

In the general population, DOACs may also lead to prolongations in the INR [8,9]. The sensitivity of the INR for DOAC depends on the type of DOAC (with rivaroxaban having the most and apixaban the least profound effect) and the reagent and coagulation analyzer used. The prolongation of the INR has a poor correlation with the plasma concentration of the drug. The INR, therefore, cannot be used to ascertain whether a clinically relevant DOAC dose is present within a patient sample as the INR may be normal in the presence of therapeutic drug doses. Conversely, the INR may also be substantially prolonged in the presence of (sub)therapeutic drug doses. It has not been studied to what extent DOACs increase the INR in patients with cirrhosis, who may already have INR prolongations at baseline.

Here, we studied the effect of DOACs on INR prolongation in patients with cirrhosis. We used samples from patients undergoing liver transplantation to which we added DOACs in clinically relevant concentrations. Specifically, we used median peak DOAC values that have been reported in patients without underlying liver disease. In addition, we studied INR changes in patients with cirrhosis who received one specific DOAC (edoxaban) for study purposes. We calculated the effects of INR prolongation by the various DOACs on the MELD score.

## 2 Materials And Methods

### 2.1 Patients

We studied samples from 20 adult patients undergoing liver transplantation at King’s College Hospital, London, between September 2017 and December 2017, and 20 healthy individuals recruited in the same study. Patient characteristics have been described previously [10]. Only samples taken after the induction of anesthesia were studied. The study was approved by the NRES Committee London—Westminster, Study Number 17/LO/0527. In addition, samples from 16 adult patients with cirrhosis and 16 healthy volunteers recruited in the University Medical Center Groningen, the Netherlands, who received edoxaban (60 mg once daily, administered for 7 consecutive days) were studied. Characteristics of patients and controls have been outlined previously [11]. Samples were taken twice at day 1 (baseline and 2 h after the first dose) and once on day 3 and day 7, 2 hours after ingestion of edoxaban. The study protocol was approved by the local medical ethical committee (METc 2016/226) and was registered at the Netherlands Trial Register (NTR6397). Both studies were executed in accordance with both the Declarations of Helsinki and Istanbul, and written informed consent was obtained from each subject before inclusion in both studies.

### 2.2 INR determination

INRs were measured on an automated coagulation analyzer (STACompact 3, Stago) with the use of reagents and protocols from the manufacturer. Plasma samples from the liver transplant recipients were spiked with dabigatran (175 ng/mL), rivaroxaban (250 ng/mL), apixaban (150 ng/mL), or edoxaban (200 ng/mL). All drugs were purchased from Alsachim (Illkirch-Graffenstaden, Germany).

### 2.3 Contribution of INR increase to the MELD score

We calculated the contribution of the INR to the MELD score as 11.2 × log_e_ (INR). The contribution of the INR to the MELD score was determined in absence and presence of a DOAC, and the increase in MELD points by the DOAC is reported.

### 2.4 Statistical analyses

Data are reported as median with ranges. The relative increase in INR after the addition of a DOAC was compared between patients and controls using a Mann–Whitney U-test. Relationship between baseline INR and INR increase after the addition of a DOAC was tested using simple linear regression, and *R*^2^ values are reported. *P* values <.05 were considered statistically significant.

## 3 Results And Discussion

In 20 patients with cirrhosis undergoing liver transplantation, the INR was 1.48 [1.06-3.76] (median [range]), whereas the INR was 1.06 [0.93-1.15] in healthy volunteers. We spiked plasma samples from these patients with DOACs with a concentration found at peak level in patients by using these drugs in a therapeutic regimen [8]. The INR increased after an *ex vivo* addition of dabigatran, rivaroxaban, apixaban, and edoxaban in both patients and controls. However, the increase in patients was more pronounced than that in controls (Table 1). The increase in INR in patients resulted in a clinically relevant increase in MELD points for all drugs, with the largest increase after the addition of rivaroxaban and the smallest increase after the addition of apixaban. In patients, but not in healthy controls, the baseline INR positively correlated to the absolute INR increase (Figure).

**Table 1:** The effect of *ex vivo* addition of DOACs on the INR in healthy individuals and patients undergoing liver transplantation sampled after the induction of anesthesia.

	INR healthy individuals, median (range); fold increase	INR patients with cirrhosis, median (range); fold increase	MELD score increase in patients by the DOAC
-	1.06 (0.93-1.15)	1.48 (1.06-3.06)	
Dabigatran (175 ng/mL)	1.35 (1.22-1.50); 1.3	2.30 (1.36-7.62); 1.5	4.6 (2.8-10.2)
Rivaroxaban (250 ng/mL)	1.99 (1.74-2.47); 1.9	3.71 (2.08-12.00); 2.5	10.2 (7.3-15.3)
Apixaban (150 ng/mL)	1.16 (1.07-1.31); 1.1	1.92 (1.15-6.41); 1.3	2.9 (0.9-8.3)
Edoxaban (200 ng/mL)	1.59 (1.46-1.81); 1.5	2.79 (1.52-8.04); 1.9	7.0 (4.0-10.8)

The fold increase in INR is significantly higher in patients for all DOACs (all *P* < .001 by the Mann–Whitney U-test).

DOAC, direct oral anticoagulant; INR, international normalized ratio.

**Figure:**
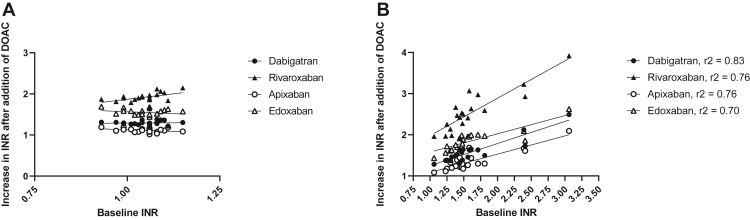
Correlation of baseline international normalized ratio values to the absolute increase in the international normalized ratio values after the addition of direct oral anticoagulants in healthy controls (A) and in patients undergoing liver transplantation (B). *R*^2^ values are indicated for patients (all *P* < .0001), and in controls, the correlations were not significant.

We next measured INRs in samples taken before and at 2 hours, 3 days, and 7 days after the start of a once daily regimen of 60 mg of edoxaban. In both patients and controls, the INR increased on edoxaban (Table 2). The increase in patients was comparable with that in controls and resulted in a median increase of approximately 5 MELD points.

**Table 2:** INR changes in healthy individuals and patients with cirrhosis before and during 1-week treatment with edoxaban.

	INR healthy individuals, median (range); fold increase	INR patients with cirrhosis,median (range); fold increase	MELD score increase in patients by the DOAC
-	0.97 (0.89-1.19)	1.11 (1.03-1.77)	
Day 1	1.43 (1.08-2.34); 1.4	1.83 (1.38-2.90); 1.6	5.6 (2.8-9.4)
Day 3	1.43 (1.18-2.41); 1.5	1.75 (1.06-2.68); 1.5	4.7 (0.2-8.5)
Day 7	1.54 (1.18-2.22); 1.6	1.79 (1.46-3.01); 1.6	5.6 (2.9-9.2)

DOAC, direct oral anticoagulant; INR, international normalized ratio.

Here, we demonstrate that *ex vivo* addition of DOACs at doses found at therapeutic peak levels in the general population to plasma from liver transplant candidates increases the INR to a greater extent compared with healthy individuals. The doses studied result in an INR prolongation that translate to a median of 3 to 10 MELD points, depending on the DOAC, and the effect is larger with a higher baseline INR. In patients with mild cirrhosis who received a therapeutic dose of edoxaban, the INR prolongation at peak levels was similar between patients and controls and resulted in an additional 5 MELD points. The clinically relevant inflation of the MELD score by peak therapeutic plasma levels call for action to fairly and adequately prioritize patients who are using DOACs while on the transplant waiting list.

A simple solution to assess INR values in patients who are using DOACs is to remove the DOAC from the blood sample before the INR determination. Commercially available agents, such as DOAC stop (Haematex), that use activated charcoal to remove DOACs from the sample have been shown to effectively neutralize DOAC effects on coagulation tests including the INR [12]. However, not all clinical laboratories offer the use of DOAC removal. Other options include the use of a simplified MELD score, as previously proposed for patients that use vitamin K antagonists while on the waiting list [7], or sampling of patients at the trough DOAC level. We have not quantified the INR effect at trough levels, but because there is a linear relation between INR and DOAC levels [8], these effects can be estimated. Because in the general population, therapeutic trough plasma levels are 5 to 10 times lower than peak levels for rivaroxaban and edoxaban, the MELD inflation is largely gone at trough levels, assuming comparable pharmacokinetics in patients with cirrhosis and individuals with a healthy liver. However, trough levels for dabigatran and apixaban are only 1.5-fold to 3-fold lower than the peak levels, which means that MELD inflation at the trough levels would still be significant. In these patients, the INR ideally could be measured after a deliberately missed dose, but this may not be feasible and safe in all patients. Importantly, because DOACs are metabolized by liver and kidney in patients with advancing cirrhosis who may be complicated by renal failure, DOAC metabolism may be compromised, leading to elevated peak and trough levels with accompanying additional elevations in INR and further inflation of the MELD score. Although DOACs are contraindicated for the sickest patients with cirrhosis, in clinical practice, DOACs may not be stopped immediately following worsening of disease, and extra care should be taken in interpreting the MELD score in such patients.

Taken together, we have demonstrated that DOACs at peak plasma levels may result in profound increases in the MELD score in patients with cirrhosis owing to their effects on the INR. These increases may still be present at trough levels in patients taking dabigatran or apixaban because plasma levels of these drugs are only 1.5-fold to 3-fold lower than the peak levels. Given the clinical relevance of our findings, confirmation of our findings by independent laboratories is warranted. Ideally, such studies would measure INRs at various time points after DOAC ingestion in larger number of patients taken all clinically available DOACs. In addition, studies assessing the strategies to determine DOAC-independent INR and MELD scores in patients on DOACs, for example, by using DOAC stop, are indicated. Given the increasing number of sicker patients who use DOACs that may continue to use these drugs while on the waiting list or even up to transplantation [[13], [14], [15]], it will be important to take DOAC use into account in the current organ allocation procedures in order to avoid an unwanted favoring of DOAC users.
